# Bisphenol S fetal exposure in ewes exhibiting contrasted metabolic status impaired the ovarian follicular development of fetuses and lambs

**DOI:** 10.1186/s13048-026-02182-y

**Published:** 2026-06-30

**Authors:** Ophélie Téteau, Marie-Emilie Lebachelier de la Riviere, Hans Adriaensen, Olivier Lasserre, Marlène Lacroix, Laetitia Corset, Peggy Jarrier-Gaillard, Pascal Papillier, Alice Desmarchais, Sandrine Freret, Virginie Maillard, Véronique Gayrard, Nicole Picard-Hagen, Sebastien Elis

**Affiliations:** 1 INRAE, CNRS, Université de Tours, PRC, Nouzilly, 37380 France; 2 INRAE, Université de Tours, CHU de Tours, PIXANIM, Nouzilly, 37380 France; 3 INRAE, PAO, Nouzilly, 37380 France; 4 Univ Toulouse, ENVT, INRAE, InTheRes, Toulouse, France; 5 Univ Toulouse, EI Purpan, ENVT, INRAE TOXALIM, Toulouse, France

**Keywords:** Endocrine disruptors, Bisphenol S, Follicular development, Obesogenic, Ewe, Transgenerational, Magnetic resonance imaging

## Abstract

The size of the ovarian reserve, defined by the pool of primordial follicles, is established during fetal life in women and sheep. The prenatal period is a critical window of sensitivity, especially for ovarian development. Maternal metabolic status and nutrition during gestation can influence fetal programming, and affect the establishment of ovarian reserve. In addition, the developing fetus is particularly sensitive to endocrine disruptors. Bisphenol A (BPA), a plasticizer widely used in food packaging, has deleterious effects on fetal folliculogenesis. BPA has been banned from the food industry and has been replaced mainly by bisphenol S (BPS), whose effects on ovarian development remain poorly characterized.

The objective of this study was to determine the effects of *in utero* exposure to BPS (50 µg/kg/day) on ovarian development in female fetuses, and lambs, using histology and/or magnetic resonance imaging (MRI). In addition, pregnant ewes exhibited contrasted metabolic status (lean versus fat), allowing assessment of the interaction between maternal nutrition and BPS exposure. This study was divided into two experiments. In the first, pregnant ewes received daily subcutaneous injection of BPS for 3 months, and fetal ovarian development was analyzed at 130 days of gestation. In the second, pregnant ewes were exposed daily to BPS through food for 3 months and female lambs were then monitored up to 4-month-old (pre-puberty). In offspring of fat mothers, the body weight of the fetuses increased but this increase was not observed anymore in lambs. Plasma anti-Mullerian hormone (AMH) concentrations decreased in 1-month-old lambs and the antral follicle number increased in both fetuses and 4-month-old lambs from well-fed mothers. In addition, a significant interaction between maternal metabolic status and gestational BPS exposure was reported for the numbers of primordial, intermediate and total follicles of fetuses, with a similar trend for pre-antral follicles. Such significant interaction was also reported for the number of intermediate follicles in 4-month-old lambs, with a similar tendency being reported for AMH concentration.

In conclusion, maternal nutrition and gestational exposure to BPS had deleterious consequences on the body growth and ovarian follicular development in the offspring. In addition, BPS effects differ according to the maternal metabolic status. Thus, the maternal environment affected folliculogenesis in both fetuses and lambs. Further research is needed to determine whether these alterations have long-term deleterious effects on their reproductive function in adulthood.

**Supplementary Information:**

The online version contains supplementary material available at https://doi.org/10.1186/s13048-026-02182-y.

## Introduction

The concept of Developmental Origin of Health and Disease (DOHaD) considers the peri-conceptual and prenatal maternal environment to play an important role in the short- and long-term health of offspring. During organogenesis, the development of the fetus in an unfavorable uterine environment can induce irreversible adaptations in the placenta and developing organs. However, in adulthood, if their post-natal environment changes, their organism may not be adapted, thereby increasing susceptibility to non-communicable diseases (obesity, type II diabetes, cardiovascular diseases, etc.) [21, 24, 34]. Consequently, the prenatal period represents a window of developmental vulnerability.

In female, the formation of primordial follicles during fetal life determines the size of the ovarian reserve, which is established before birth in both women and ewes [37]. Thus, any abnormalities occurring during gestation could have long-term consequences on the reproductive capacity of the offspring. Maternal nutrition and metabolic status during gestation are among the most important parameters that influence fetal programming [5, 21, 43]. Maternal malnutrition (undernutrition or overweight / obesity) currently affects approximately one-third of the world population [34]. Maternal nutrition can alter the synthesis of endogenous maternal hormones that are critical for normal fetal development [12]. In animal models, maternal overnutrition during gestation decreased the number of primordial follicles in adult mouse offspring [7], whereas undernutrition decreased the ovulation rate of the ewe and cow offspring [12, 14]. Thus, altered maternal nutrition status during gestation has deleterious consequences on the follicular development of the offspring, which can affect their reproductive function in adulthood.

Neonatal ovarian development is also highly sensitive to estrogens [28]. Some endocrine disruptors (EDCs) have oestrogenomimetic properties. The fetus and the developing young are particularly sensitive to these molecules, since the mechanism of hepatic biotransformation and detoxification are immature [3]. Due to its well-documented deleterious effects on health [26, 31], BPA use was regulated at a dose of 50 µg / kg / d in 2006, then was banned from food contact packaging in 2015 in Europe [13]. However, BPA has largely been replaced by bisphenol S (BPS), a structural analogue. Unregulated and unrestricted for use, BPS is now also found in biological fluids (urine (2.6 nM), placenta (24.4 pg / g), breast milk (1.5 nM) [11, 40, 50]. In ovine model, BPS was able to cross the placental barrier and accumulate in the fetal compartment [18]. In mice, gestational exposure to BPS 50 µg / kg / day decreased the pool of primordial follicles [47], while exposure to BPS 2 and 10 µg / kg / day increased the number of primordial follicles in offspring [58]. Thus, despite dose-dependent divergent effects, exposure to BPS during gestation would disrupt the primordial follicle pool of the offspring, which could decrease their reproductive longevity. To date, only few studies have investigated the impact of BPS on follicular development *in utero*, and the mechanisms underlying its reproductive toxicity remain to be investigated. Maternal metabolic status and exposure to BPS during gestation have each been shown to adversely affect ovarian development in offspring. In addition, the prevalence of metabolic disorders [34] and environment exposure to BPS [54] are both constantly increasing worldwide. We therefore hypothesize that the metabolic status could potentiate the adverse effects of BPS exposure during gestation, as previously reported for others EDCs such as phthalates in mice [25]. In the ovine model, significant interactions between BPS exposure and metabolic status have already been reported as affecting the ovary, in terms of oocyte quality [10], ovarian follicular steroidogenesis [51] and granulosa cell proteome [27], whereas such interactions were not observed in other tissues such as the oviduct proteome [32] or the hypothalamus mRNA expression [9].

The objective of this study was to investigate the effects of maternal metabolic status and/or exposure to BPS from 1 to 4 months of gestation on the pre- and post-natal development in the ovine offspring. This period corresponds to the development and organogenesis of the fetus, a sensitive window of exposure to endocrine disruptors [33]. Fetuses and offspring originating from pregnant ewes with contrasted metabolic status were monitored for body weight and ovarian development from the fetal stage to the pre-puberty stage. Ovarian morphology was assessed using Magnetic Resonance Imaging (MRI), a relevant tool for this analyzis [60]. In addition, Anti-Müllerian Hormone (AMH) concentrations, a marker of ovarian reserve, were evaluated [35]. Finally, we categorized, counted and analyzed all stages of folliculogenesis through histology analyses of the ovary.

## Materials and methods

### Ethics

All experimental procedures were conducted in accordance with the European Directive 2010/63/EU on the protection of animals used for scientific purposes and approved by the French Ministry of National Education, Higher Education, Research and Innovation after ethical assessment by the local ethics committee: “Comité d’Ethique en Expérimentation Animale TOXCOM 0253” (protocols registered under APAFIS number 23515-2020010814364558_v4) for the experiment with fetuses and “Comité d’Ethique en Expérimentation Animale Val de Loire (CEEA VdL)” (protocols registered under APAFIS numbers 2020030611546813_v1 and 2020030611343222_v2) for the experiment with lambs. All animals originated from INRAE experimental Unit “Unité expérimentale Physiologie Animale de l’Orfrasière” and INRAE consented to use the animals in these experimental protocols.

### Experimental design, nutritional management of pregnant ewes

Two complementary experimental designs were carried out to investigate the effects of gestational exposure to bisphenol S (BPS) on ovarian development. The first experiment focused on fetal outcomes, and the second experiment assessed post-natal effects.

**Fig. 1:**
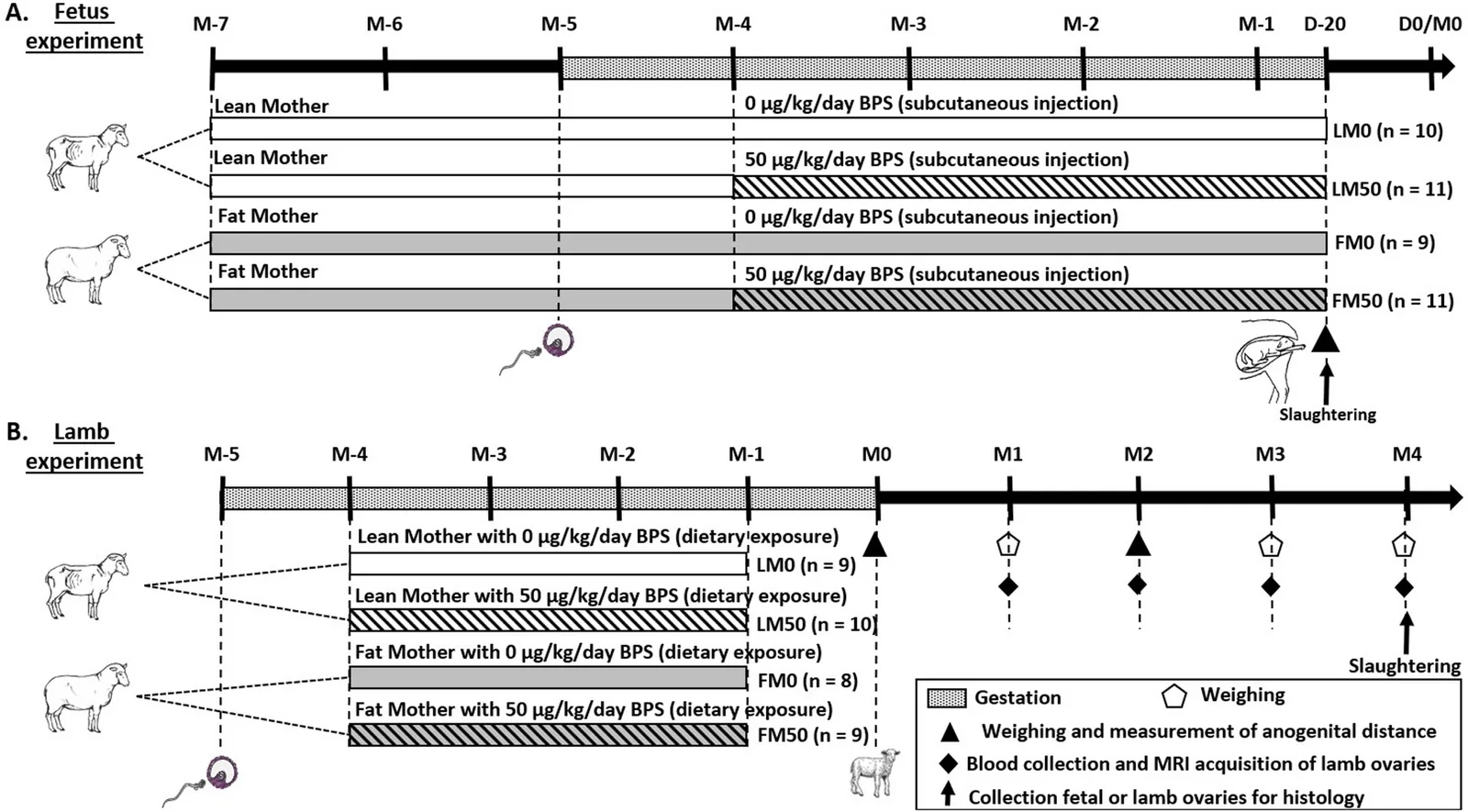
Experimental design of the in utero BPS exposure of fetus (A) and lambs (B)

In the first experiment (Fig. 1A), Ile-de-France ewes (*n* = 41) were transferred and housed in the experimental unit of the Toulouse National Veterinary School (France). The animals were free to interact with each other. The ewes (age mean 2.3 years at 30 days of gestation) were distributed into two diet groups to induce contrasting metabolic statuses: lean mother ewes (LM; 51.94 ± 1.23 kg at around 30 days of gestation) and fat mother ewes (FM; 66.58 ± 1.43 kg at around 30 days of gestation). The diet was distributed twice daily: the lean mother ewes received 350 g/d of a feed composed of 25% crude protein, while the fat mother ewes received 1 kg/d of a feed composed of 18% crude protein (Brebilac^®^) at the beginning of the experiment. Feed rations were adjusted every 14 days, based on body weight gain throughout gestation. All ewes had ad libitum access to straw and received a mineral-vitamin supplement (20 g / d, ALIMAL GESTANTE^®^ granules, 10% Potassium, 6% Calcium, 6% Magnesium). Feed rations began two months prior to mating (M-7), during the breeding season, with M0/D0 corresponding to the birth of offspring.

After 30 days of gestation (M-4), ewes from each metabolic group were subdivided according to BPS exposure and received daily subcutaneous injection of either vehicle or BPS (50 µg BPS / kg / d). Thus, four experimental groups were constituted: lean mother LM0 (*n* = 10) and fat mother FM0 (*n* = 9) control ewes, and LM50 (*n* = 11) and FM50 (*n* = 11) BPS-exposed ewes. The BPS dose was selected based on the tolerable daily intake previously established for BPA in Europe in 2006 [13]. Exposure continued until day 130 of gestation (D − 20), corresponding to the end of the experiment. Blood samples were collected from ewes before (M-4) and after (D-20) BPS exposure. At 130 days of gestation (D-20), dams were euthanized by intravenous pentobarbital overdose (60 mg/kg of Dolethal^®^ Vetoquinol, Lure, France). After uterine extraction, fetuses were weighted and fetal blood sample was collected by direct intracardiac puncture, just before its euthanasia by intracardiac overdose of pentobarbital (60 mg/kg, Dolethal^®^, Vetoquinol SA, Lure, France). Anogenital distance (AGD) was measured to assess the degree of masculinization, and fetal ovaries as well as blood samples were collected. A total of 37 female fetuses were collected during this experiment (LM0 *n* = 7, LM50 *n* = 11, FM0 *n* = 11, and FM50 *n* = 8), but the anogenital distance were measured on 32 fetuses.

In the second experiment (Fig. 1B.), Ile-de-France ewes (*n* = 36; age mean 3.4 years-old) were group-housed in a sheepfold at the INRAE Experimental Unit PAO (Nouzilly, France). During the breeding season, two groups of primiparous ewes exhibiting contrasted metabolic status, called Lean Mother (LM) with a weight < 72 kg (60.05 ± 1.48 kg) and Fat Mother (FM) with a weight > 72 kg (82.94 ± 1.50 kg), were formed and were subsequently mated with rams. All ewes received straw *ad libitum* and a dietary supplement (AXEREAL Elevage, saint Germain de salles, France), composed of wheat (60%), alfalfa, sugar cane treacle and vitamins, distributed once a day in the morning all along the experiment (ewes from each experimental group were housed in the same enclosure and received food at the same time). LM ewes received 94 g/d of the dietary supplement and FM ewes received 124 g/d of the same supplement.

Within each metabolic group, pregnant ewes were then subdivided according to their dietary BPS exposure at a dose of 50 µg BPS / kg / d, BPS or vehicle being mixed in the diet. Thus, four experimental groups of pregnant ewes were established: LM0 (*n* = 9) and FM0 (*n* = 8) as not exposed controls, and LM50 (*n* = 10) and FM50 (*n* = 9) exposed to 50 µg BPS/kg/day. Since M0/D0 refers to the birth of the offspring, the ewes received this treatment between 1 (M-4) and 4 (M-1) months of gestation. Exposure to BPS did not start from the mating, to avoid potential effects on oocyte quality, embryo development or implantation [56]. The ewes were weighed during the third month of gestation, in order to adjust feed intake. Blood samples were collected before (M-4 and after (M-1) exposure to BPS.

At birth (M0/D0), the lambs were separated from their mother and reared on artificial milk. Lambs were weighed once a month from birth (M0/D0) to 4-month-old (M4), and anogenital distance was measured at birth (M0/D0) and 2 months later (M2). In addition, from 1 to 4 months, the female lambs had a monthly pelvic magnetic resonance imaging (MRI) to analyze the follicular population of the ovaries, and blood samples were collected. At the prepubertal stage (M4), lambs were rendered unconscious by electronarcosis then bled in the INRAE experimental slaughterhouse (Nouzilly, France) to be sacrificed. Ovaries were collected and weighed. The liver and kidneys were also weighed. Organ weight (g) was adjusted with body weight (kg) to account for inter-individual variability. A total of 36 female lambs were included in this experiment (LM0 *n* = 7, LM50 *n* = 11, FM0 *n* = 9, and FM50 *n* = 9).

In this paper, two experiments were performed to determine the effects of *in utero* BPS exposure on fetuses and lambs. In fetus experiment (A.), adult ewes were fed a diet to induce either a lean (LM) or fat (FM) metabolic status, starting 2 months (M-7) before breeding until the end of the experiment. During the breeding season, these ewes were mated with rams (M-5). The gestation length in ewes is 150 days. After 1 month of gestation (M-4) and until the end of the experiment (D-20), the pregnant ewes received a daily subcutaneous injection of BPS at either 0 µg / kg / day or 50 µg / kg / d. Four experimental groups were thus established: lean without BPS (LM0; *n* = 10), lean with BPS 50 µg/kg/d (LM50; *n* = 11), fat without BPS (FM0; *n* = 9), and fat with BPS 50 µg/kg/d (FM50; *n* = 7). At 130 days of gestation (D-20), the animals were sacrificed and female fetuses were weighed, anogenital distance was measured, and one ovary was fixed and embedded in paraffin for histological analyses. A total of 37 female fetuses were born during this experiment (LM0 *n* = 7, LM50 *n* = 11, FM0 *n* = 11, and FM50 *n* = 8).

In lamb experiment (B.), adult ewes were divided into two groups according to their metabolic status. Ewes with a weight < 72 kg were classified as lean ewes (LM) and ewes with a weight > 72 kg were classified as fat ewes (FM). During the breeding season, these ewes were mated with rams (M-5). After 1 month of gestation (M-4) and until 4 months of gestation (M-1), the pregnant ewes received BPS in their diet of BPS either at 0 µg / kg / day or 50 µg / kg / d. Four experimental groups were established: lean without BPS (LM0; *n* = 9), lean with BPS 50 µg/kg/d (LM50; *n* = 10), well-fed without BPS (FM0; *n* = 8), and well-fed with BPS 50 µg/kg/d (FM50; *n* = 9). From birth (D0) to 4 months (M4), the female lambs were weighted, and anogenital distance was measured at birth (D0) and 2 months (M2). In addition, from 1 to 4 months, the female lambs underwent pelvic Magnetic Resonance Imaging (MRI) for ovarian assessment, as well as a blood collection. At 4 months (M4), the female lambs were sacrificed and one ovary was fixed and embedded in paraffin for histological analyses. A total of 36 female lambs were included in this experiment (LM0 *n* = 7, LM50 *n* = 11, FM0 *n* = 9, and FM50 *n* = 9).

### Ewe plasma BPS-glucuronide (BPS-g) assays

Blood samples (5 mL) were collected on pregnant ewes by jugular venipuncture, in heparinised tubes (17 IU/mL sodium heparin, Vacutainer^®^; Becton Dickinson and Company, Le Pont de Claix, France). Sampling were performed 24 h after the last BPS injection for ewes in the fetal experiment and 1 h after the last dietary administration for ewes in the lamb experiment, at the end of the exposure period (D-20 / M-1, respectively). Blood samples were centrifuged (3,700 g for 30 min at 4 °C), and plasma were stored at -80 °C until analysis.

BPS and BPS-g concentrations were quantified in ewe plasma on the same analytical run, as previously explained [10]. Briefly, the concentrations were quantified using ultra-high-performance liquid chromatography coupled to tandem mass spectrometry with an Acquity U-HPLC device coupled to a Xevo-TQ triple quadrupole mass spectrometer (Waters, Saint-Quentin-en-Yvelines, France) operated with positive electrospray ionisation and MRM mode. The limit of quantification (LOQ) was set at 0.5 ng/mL (2 nM) for BPS and 0.05 ng/mL (0.10 nM) for BPS-g.

### Female lamb plasma anti-müllerian hormone assay

Blood samples (2.5 mL) were collected on female lambs by jugular venipuncture, in heparinized tubes (17 IU/mL sodium heparin, Vacutainer^®^; Becton Dickinson and Company, Le Pont de Claix, France), once a month from 1 to 4 months (M1, M2, M3 an M4). These blood samples were centrifuged (3,700 g for 30 min at 4 °C), and plasma was stored at -20 °C until AMH assays.

Plasma AMH concentrations were determined using the AMH Gen II ELISA assay (Beckman Coulter, Villepinte, France), according to the manufacturer’s instructions, and already validated for the analysis of sheep samples [38]. Briefly, AMH concentrations were determined in 50 µL-undiluted plasma. The limit of detection for this assay was 78 pg/mL, and the intra-assay coefficients of variation was lower than 7%. AMH assays were also performed on fetal plasma, but AMH concentrations were below the limit of detection in all fetal samples.

### MRI acquisition

To quantify follicles ≥ 1 mm in diameter, 5 female lambs per group underwent pelvic MRI at 1, 2, 3 and 4 months (M1, M2, M3 and M4) using a 3T Magnetom^®^ Verio MRI scanner (Siemens, Erlangen, Germany) on the “Phénotypage par Imagerie in/ex vivo de l’Animal à la Molécule” (https://pixanim.val-de-loire.hub.inrae.fr/ PIXANIM, INRAE, Nouzilly) platform. This device ensured high-quality images for all experimental groups. First, lambs were anaesthetized intravenously (i.v.) with 0.1–0.4 mg/kg xylazine (ROMPUN^®^ 2%, Bayer Division Animal Health, La Garenne-Colombes, France) and 5–10 mg/kg ketamine (IMALGENE^®^, Merial, Lyon, France) followed by inhalation maintenance anaesthesia with 3% isofluorane (Piramal, India). For the in vivo ovary image acquisition, two Radio Frequency (RF) coils were used: a spine antenna allowing the lamb to be laid on it with a sphynx position and a second body matrix antenna covering the back of the lamb Those two antennas were thus positioned on each side of the lamb’s pelvis under investigation. Three-dimensional MRI acquisitions were performed with a T2 (transverse relaxation time) image contrast. This 3D T2 SPACE (Sampling Perfection with Application-optimized Contrasts) allowed a high contrast of follicles within the ovary thanks to their high liquid rate, related to their antrum filled with follicular fluid.

The MRI acquisition parameters were set as follows: Repetition Time (TR) = 1500 ms, Echo Time (TE) = 155 ms, flip angle = 140 degrees, Echo Train Length (ETL): 422 and Turbo Factor: 71. The Field-of-View (FOV): was 320 × 320 mm², matrix: 768 × 768² and slice thickness: 0.4 mm (with 352 slices), resulting in a voxel size of 0.4 × 0.4 × 0.4 mm^3^. The inter-echo spacing was 7.4 ms. A bandwidth of 283 Hz/Px was used. Further, only one Number of Excitation (NEX) and an integrated Parallel Acquisition Technique (iPAT) of 3 was used producing a total acquisition time of 41 min 38 s.

For analyses, the Digital Imaging and Communication in Medicine (DICOM) images were converted to the Neuroimaging Informatics Technology Initiative (NIfTI) format. These analysis were carried out with the ITK-SNAP software version 3.8.0 (www.itksnap.org*)* [57]. A stack of successive images from the SPACE MRI sequence covering the entire ovary were analysed. Thus, size (mm) and ovarian volume (mm^3^) were determined for both ovaries of each lamb. Antral follicles appeared as white ovoid structures. Thus, the number, size (mm) and volume (mm^3^) of each follicle ≥ 1 mm could be assessed by colorising each follicle and thus ending up with the entire ovaries to be segmented. The size was determined by averaging the height and width of the maximum flat section of the ovary and of the follicles (Supplementary Fig. 1). The results are expressed as the mean ± SEM. Because a high number of follicles can be indicative of a pathology (a woman ovary is considered polycystic with a number of follicles per ovary ≥ 25), the percentage of lambs exhibiting ≥ 25 follicles in each ovary was recorded. The results are expressed as the percentage ± SEM of 5 lambs per experimental group.

### Ovary histology

At the end of the experiment (D-20) for fetuses and at 4 months (M4) for lambs, animals were sacrificed and one ovary per lamb and fetus was fixed in 4% paraformaldehyde (PAF) for histologic analyses. Ovaries were embedded in paraffin and serially sectioned to a thickness of 7 μm with a microtome. Two sections, located within the central region and spaced at least ten sections apart to avoid counting the same follicle twice, were stained with hematoxylin. Slides were scanned, using slide scanner Axioscan Z1-ZEISS, at 10X magnification, with a resolution of 0.44 μm / pixel. Subsequently, the follicles were counted with the QuPath-0.2.3 software (University of Edinburgh, United Kingdom; https://qupath.github.io/). The follicles were categorized based on the shape and number of somatic cell layers surrounding the oocyte, as well as the presence or absence of an antrum. Thus, the follicles were organized into six classes: primordial follicle with only flattened granulosa cells (GC) around the oocyte, intermediate follicle with at least one rounded GC around the oocyte, primary follicle with a layer of rounded GC around the oocyte, secondary follicle with two layers of GC, pre-antral follicle with more than two layers of GC, and antral follicle with the presence of an antrum. (Supplementary Fig. 2). Only follicles with a visible oocyte were counted, in order to ensure that the same follicle was not counted across multiple sections. Results are expressed as the number of follicles of each class normalized to the surface of the section (mm^3^), as the mean ± SEM of 7–9 fetuses or 6–10 lambs per experimental group.

### Statistical analysis

Statistical analyses were performed with the RStudio software (RStudio Team (2020). RStudio: Integrated Development for R. RStudio, PBC, Boston, MA URL http://www.rstudio.com/, version 2024.09.1 + 394).

Ewes bodyweight at 1 month of gestation, plasma BPS and BPS-g concentration, number of fetus and lamb per ewe and gestation duration were presented as mean ± SEM and analysed using linear models (lm() function in R). Lamb bodyweight, plasma AMH concentration, anogenital distance, volume and size of lamb ovaries were presented as mean ± SEM and analysed using linear mixed models with repeated measures (lme() function from the nlme package). As measurements were repeated for the same individual at different times, a random term was included to model intra-individual correlation. Fetus bodyweight and anogenital distance, lamb organ weight (liver, kidney and ovary), size and volume of ovarian follicles were presented as mean ± SEM and analysed with linear mixed models using the lme() function from the nlme package in R. Although no repeated measurements were made over time, a random effect was incorporated into the model to account for the hierarchical structure of the data, including individuals from the same litter. Diet, BPS exposure and diet-by-BPS exposure interaction were assessed, as well as the litter effect for the lamb and fetus bodyweight and follicle number. Tukey’s post hoc test (multcomp package) was executed to determine differences between experimental groups. Significant difference was indicated when p-value ≤ 0.05, and a tendency when 0.05 < *p* ≤ 0.1.

## Results

### Effects of BPS on pregnant ewes

The body weight of the ewes was measured at around 1 month of gestation, before BPS exposure. In the “fetus” experiment, the difference in weight generated by diet was around 15 kg (metabolic effect (metab effect), *p* < 0.001) between lean mothers (LM) (*n* = 21, 51.94 ± 1.23) and fat mothers (FM) (*n* = 20, 66.58 ± 1.43). In the “lamb” experiment, the difference in weight between LM (*n* = 19, 60.05 ± 1.48 and FM (*n* = 17, 82.94 ± 1.5) was around 22 kg (metab effect, *p* < 0.001) (Table 1).

At 4 months of gestation, maternal plasma concentrations of BPS-glucuronide (BPS-g), a marker of internal BPS exposure, were 5.80 ± 0.82 nM in “fetus” experiment (detected in 100% of the 22 LM50 and FM50 ewes), 24 h after the last administration, and 199.63 ± 8.46 nM in the “lamb” experiment (detected in 100% of the 19 LM50 and FM50 samples), 1 h after the last distribution of the BPS-containing diet in the “lamb” experiment (Table 1). None of the 21 control ewes of the “fetus” experiment exhibited BPS-g and only one control ewe of the “lamb” experiment showed plasma BPS-g at a concentration close to the limit of detection. These results confirm effective exposure to BPS in LM50 and FM50 pregnant.

In both experiments, more than half of the ewes gave birth to twins or > 2 fetuses or lambs. The total number of fetuses or lambs, the number of males or females, or the gestation length (*n* = 36, 147.72 ± 0.25 d) were not affected by the metabolic status of the ewes and / or exposure to BPS. However, there was a tendency for the number of males to decrease following BPS exposure in the lamb experiment (*p* = 0.099) but not in the fetus experiment (*p* = 0.52) (Table 1).

**Table 1:** Characterization of ewes and their gestation. Linear regression *p*-values are presented for the effects of diet, dietary exposure to BPS and interaction of these effects. Bold text indicates significant differences (*p* < 0.05), and italicised text indicates tendencies (0.05 < *p* < 0.1)

Parameters	Mean ± SEM				*p*-value		
	LM0	LM50	FM0	FM50	BPS effect	Metab effect	BPS x Metab effect
*Fetus experiment*	*n* = 10 ewes	*n* = 11 ewes	*n* = 9 ewes	*n* = 11 ewes			
Ewe body weight (kg) at 1 month of gestation	51.92 ± 1.24^**a**^	51.96 ± 2.14^**a**^	67.69 ± 2.36^**b**^	65.67 ± 1.80^**b**^	0.622	**< 0.001**	0.596
Ewe plasma BPS-G (nM ; 24 h post BPS)	NA	5.7 ± 1.36	NA	5.9 ± 0.98	NA	0.908	NA
Number of fetuses per ewe	1.8 ± 0.2	1.64 ± 0.2	2.11 ± 0.26	1.82 ± 0.18	0.302	0.257	0.760
Number of female fetuses per ewe	0.70 ± 0.21	1.00 ± 0.23	1.22 ± 0.32	0.73 ± 0.24	0.734	0.713	0.122
Number of male fetuses per ewe	1.10 ± 0.18	0.64 ± 0.15	0.89 ± 0.20	1.09 ± 0.28	0.516	0.507	0.128
*Lamb experiment*	*n* = 9 ewes	*n* = 10 ewes	*n* = 8 ewes	*n* = 9 ewes			
Ewe body weight (kg) at 1 month of gestation	58.85 ± 2.07^**a**^	61.12 ± 2.16^**a**^	84.00 ± 2.51^**b**^	82.00 ± 1.84^**b**^	0.906	**< 0.001**	0.329
Ewe plasma BPS (nM ; 1 h post-BPS)	0.28 ± 0.28^**a**^	2.58 ± 0.24^**b**^	0.52 ± 0.52^**a**^	3.49 ± 0.92^**b**^	**< 0.001**	0.283	0.545
Ewe plasma BPS-G (nM ; 1 h post-BPS)	0.14 ± 0.14^**a**^	187.79 ± 14.15^**b**^	0.00 ± 0.00^**a**^	212.79 ± 6.97^**b**^	**< 0.001**	0.122	0.157
Gestation duration (d)	147.56 ± 0.65	147.20 ± 0.44	148.38 ± 0.26	147.89 ± 0.51	0.406	0.141	0.896
Number of lambs per ewe	2.00 ± 0.17	1.90 ± 0.31	2.88 ± 0.48	1.89 ± 0.31	0.124	0.222	0.185
Number of female lambs per ewe	1.00 ± 0.29	1.10 ± 0.41	1.25 ± 0.31	1.00 ± 0.37	0.856	0.856	0.627
Number of male lambs per ewe	1.00 ± 0.24	0.80 ± 0.29	1.62 ± 0.26	0.89 ± 0.26	*0.099*	0.210	0.323

*Metab* Metabolic status, *LM0* Lean mother unexposed, *LM50* Lean mother with BPS 50 µg / kg / d, *FM0* Fat mother unexposed, *FM50* Fat mother with BPS 50 µg / kg / d

Bold text indicates significant differences (*p* < 0.05), and italicised text indicates tendencies (0.05 < *p* < 0.1), different letters (a, b) indicate significant difference between groups (p < 0.05)

### Effects of *in utero* BPS exposure on fetus and lamb body and organ weights

**Fig. 2:**
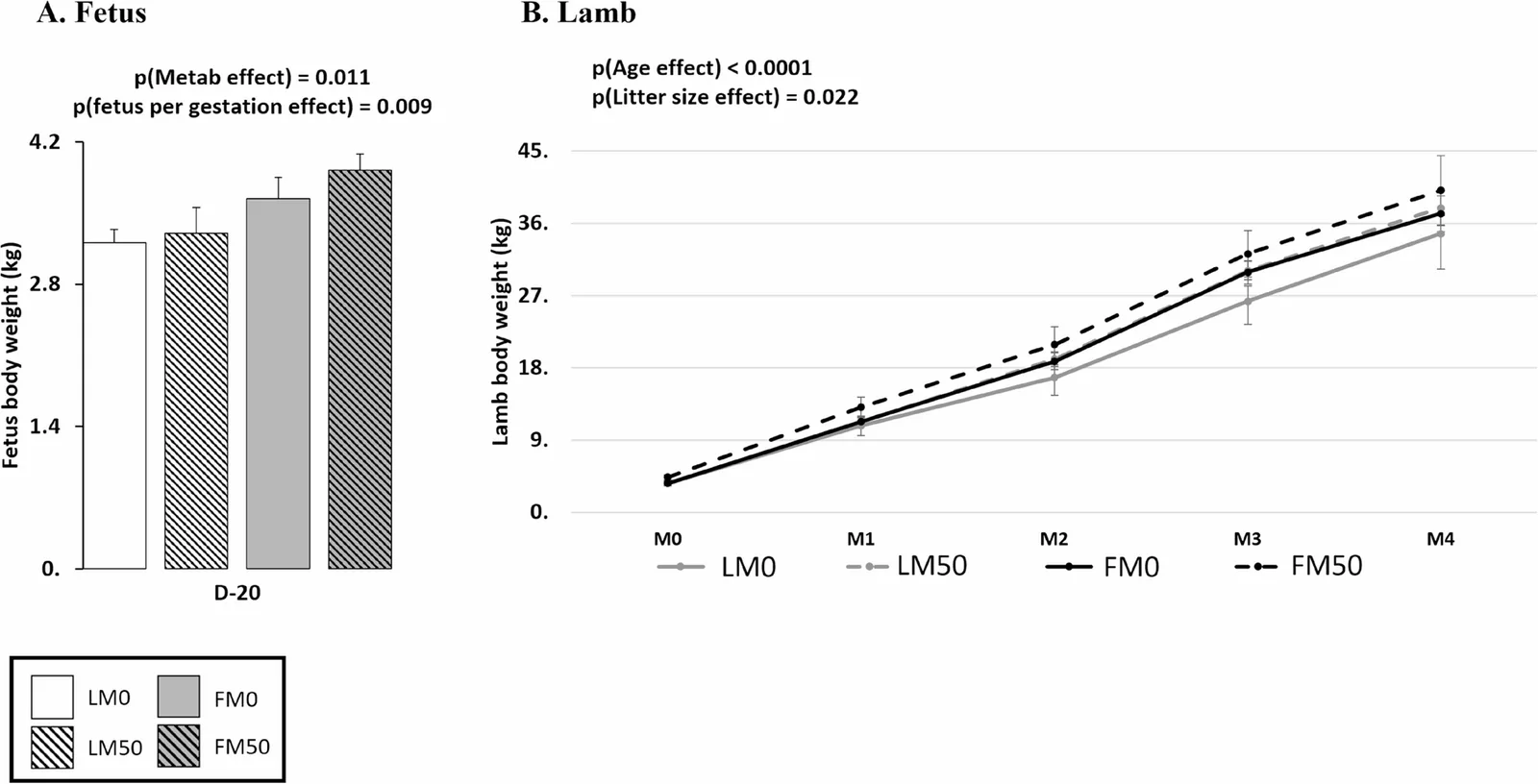
Weight of female fetus (A) and lambs (B) from ewes with metabolic status contrasted and exposed to BPS during pregnant

Body and organ weights were analyzed for females, taking into account the effect of litter size (Tables 2 and 3). At 130 days of gestation (D-20, Fig. 2A), female fetuses from lean mothers had a lower weight than those from fat mothers (3.30 ± 0.09 kg vs. 3.77 ± 0.07 kg, respectively; metab effect, *p* = 0.011). As expected, fetal bodyweight decreased with litter size (litter size effect, *p* = 0.009, 4.46 ± 0.37 kg for singleton gestation vs. 3.47 ± 0.12 kg for twin gestation vs. 3.33 ± 0.27 kg for triplet gestation, respectively) and was not being affected by BPS exposure (*p* = 0.327).

**Table 2:** Body weight and number of total follicle of fetuses and lambs according to gestation

Parameters	Mean ± SEM					*p*-value			
	Single	Double	Triple	Quadruple	Sextuple	BPS effect	Metab effect	BPS x Metab effect	Litter size effect
*Fetus experiment at M-1*	*n* = 3 fetus	*n* = 23 fetus	*n* = 7 fetus						
Bodyweight (kg)	4.46 ± 0.37 ^a^	3.47 ± 0.12 ^b^	3.33 ± 0.27 ^b^	-	-	0.327	**0.011**	0.527	**0.009**
Number of follicle/mm^2^	186 ± 23.7	138 ± 7.03	84.9 ± 20.0	-	-	0.127	**0.015**	**0.009**	0.192
*Lamb experiment*	*n* = 3 lamb	*n* = 17 lamb	*n* = 6 lamb	*n* = 7 lamb	*n* = 2 lamb				
Bodyweight (kg) at M0	4.52 ± 0.19 ^ac^	4.34 ± 0.20 ^ac^	3.32 ± 0.29 ^ac^	2.91 ± 0.23 ^bc^	3.26 ± 0.27 ^ac^	*0.097*	*0.090*	0.584	**0.0001**
	*n* = 3 lamb	*n* = 13 lamb	*n* = 7 lamb	*n* = 5 lamb	*n* = 1 lamb				
Number of follicle/mm^2^ at M4	5.83 ± 0.25	3.24 ± 0.62	3.45 ± 1.05	3.94 ± 2.18	3.96	0.837	0.535	*0.091*	0.654

*Metab* Metabolic status

Bold text indicates significant differences (*p* < 0.05), and italicised text indicates tendencies (0.05 < *p* < 0.1), different letters (a, b) indicate significant difference between groups (p < 0.05)

**Table 3:** Organ/body weight ratio of lambs from ewes with contrasted metabolic status and exposed to BPS during pregnancy

Parameters	Mean ± SEM				*p*-value		
	LM0	LM50	FM0	FM50	BPS effect	Met effect	BPS x Met effect
*Lamb experiment*	*n* = 6 lamb	*n* = 11 lamb	*n* = 7 lamb	*n* = 6 lamb			
Ratio liverweight/bodyweight	17.9 ± 0.68	18.2 ± 0.61	17.2 ± 0.72	18.2 ± 0.92	0.399	0.516	0.300
Ratio kidneyweight/bodyweight	1.49 ± 0.04	1.46 ± 0.06	1.47 ± 0.11	1.50 ± 0.04	0.858	0.631	0.695
Ratio ovaryweight/bodyweight	0.0145 ± 0.003	0.0148 ± 0.001	0.0122 ± 0.001	0.0164 ± 0.002	0.946	0.258	0.396
Absolute ovarian weight (g)	1.05 ± 0.31	1.25 ± 0.15	1.14 ± 0.05	0.98 ± 0.15	0.907	0.652	0.388

*Metab* Metabolic status, *LM0* Lean mother unexposed, *LM50* Lean mother with BPS 50 µg / kg / d, *FM0* Fat mother unexposed, *FM50* Fat mother with BPS 50 µg / kg / d

In fetus experiment, adult ewes were fed a diet to obtain lean (LM) or fat (FM) ewes and received BPS by daily subcutaneous injection of 0 µg / kg / d or 50 µg / kg / d from 1 to 4 months gestation. Thus, four experimental groups were constituted (LM0, *n* = 7 female fetuses; LM50, *n* = 11 female fetuses; FM0, *n* = 11 female fetuses; FM50, *n* = 8 female fetuses). The body weight of the fetus was measured at the end of the experiment at 120 days of gestation (D-20) (A.). In lamb experiment, adult ewes had a contrasting metabolic status, ewes with a weight < 72 kg were classified as lean (LM), and ewes with a weight > 72 kg were classified as well-fed (FM). The ewes received BPS in their diet of 0 µg / kg / d or 50 µg / kg / d from 1 to 4 months gestation. Thus, four experimental groups were constituted (LM0, *n* = 7 female lambs; LM50, *n* = 11 female lambs; FM0, *n* = 9 female lambs; FM50, *n* = 9 female lambs). The lamb body weight was measured at birth (MO), 1 month (M1), 2 months (M2), 3 months (M3) and 4 months (M4) (B.). At the end of the experiment, at 4 months, the liver (C.), kidneys (D.) and ovaries (E.) were weighed and compared to the body weight of the lambs (g / kg). Results are presented as mean + SEM. Statistical analysis was performed between the experimental groups of a same age (D-20 or MO or M1 or M2 or M3 or M4). Bars with different letters are significantly different (*p* < 0.05).

Regarding female lambs (Fig. 2B), no differences in body weight were reported between lambs from either LM or FM (metab effect, *p* = 0.825; BPS effect, *p* = 0.275). However, as expected, body weight increased with lamb age by around 7 to 10 kg per month (age effect, *p* < 0.001, M0: 3.82 ± 0.16 kg, M1: 11.52 ± 0.41 kg, M2: 18.9 ± 0.67 kg, M3: 29.74 ± 0.89 kg, M4: 37.53 ± 1.31 kg), and decreased according to the litter size (litter size effect, *p* = 0.026, singleton gestation: 20.48 ± 3.01 kg, twin gestation: 20.9 ± 1.67 kg, triplet gestation: 19.37 ± 2.17 kg, quadruple gestation: 17.49 ± 2.19 kg, sextuple gestation: 18.46 ± 3.46 kg).

After the slaughter of lambs (M4), the weight of the liver, the organ allowing the transformation of BPS into BPS-g, and of the kidneys, organ-excreting toxins, were weighed and normalized to the bodyweight of each lamb and reported in Table 3. Normalized liver weight, average kidney weight and average ovary weight were similar between the experimental groups with no effect of the metabolic status nor BPS.

In addition, the anogenital distance of fetuses (D-20) and lambs at birth (M0/D0) and at 2 months (M2) were measured. No difference was observed between the different experimental groups (Table 4).

**Table 4:** Anogenital distance of fetus and lambs from ewes with contrasted metabolic status and exposed to BPS during pregnancy

Parameters	Mean ± SEM				*p*-value		
	LM0	LM50	FM0	FM50	BPS effect	Metab effect	BPS x Metab effect
*Fetus experiment at M-1*	*n* = 7 fetus	*n* = 8 fetus	*n* = 9 fetus	*n* = 8 fetus			
Anogenital distance (cm)	1.29 ± 0.14	1.26 ± 0.17	1.38 ± 0.11	1.35 ± 0.2	0.874	0.572	0.989
*Lamb experiment*	*n* = 7 lamb	*n* = 9 lamb	*n* = 9 lamb	*n* = 11 lamb			
Anogenital distance (cm) at M0	2.9 ± 0.33	2.84 ± 0.15	2.97 ± 0.24	3.17 ± 0.25	0.655	0.669	0.695
Anogenital distance (cm) at M2	3.58 ± 0.23	3.9 ± 0.09	3.8 ± 0.2	3.73 ± 0.17			

*Metab* Metabolic status, *LM0* Lean mother unexposed, *LM50* Lean mother with BPS 50 µg / kg / d, *FM0* Fat mother unexposed, *FM50* Fat mother with BPS 50 µg / kg / d

### MRI analysis of the effects of *in utero* BPS exposure on the ovary

**Fig. 3:**
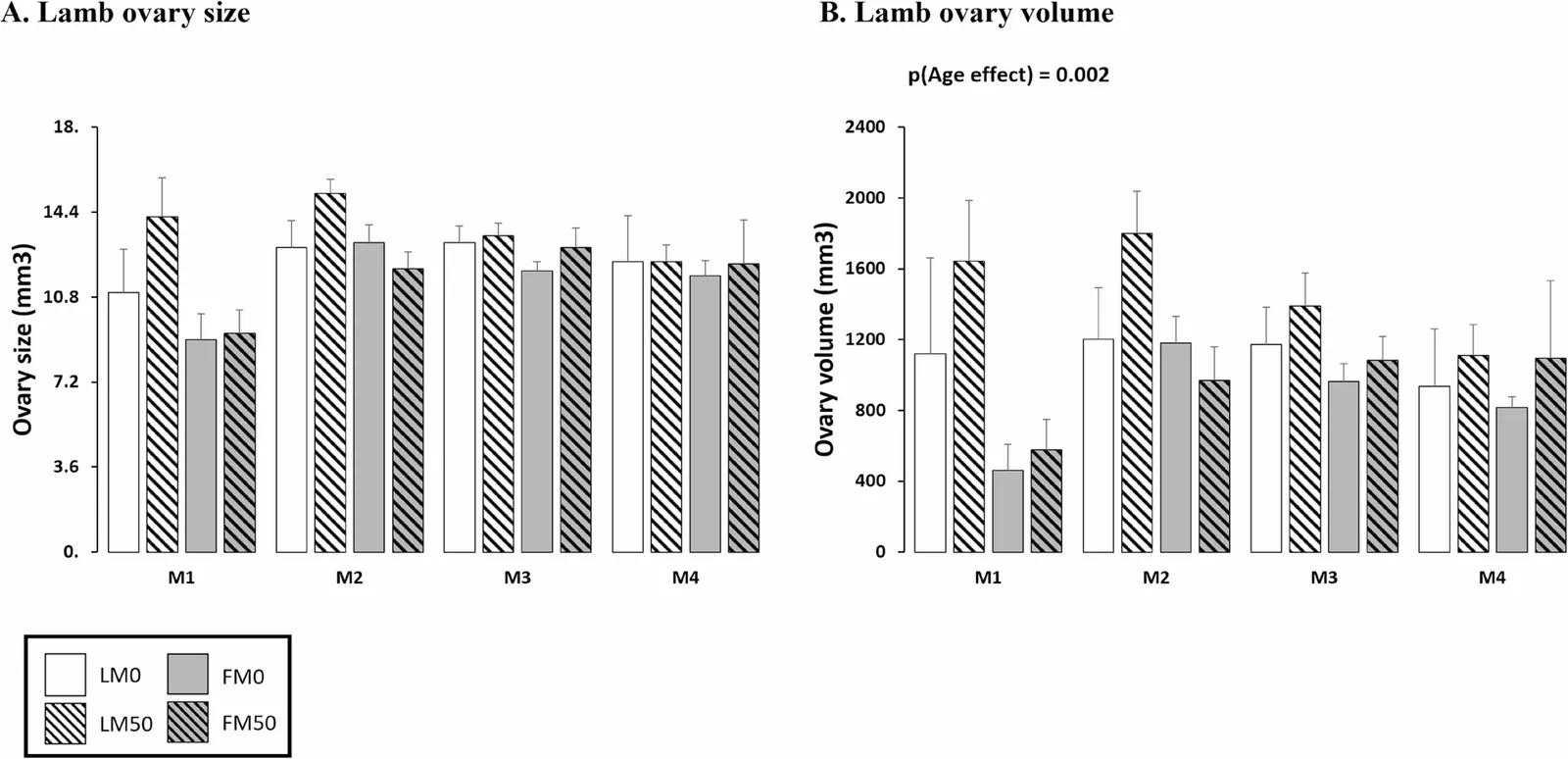
Ovarian and follicular development (A, size and B, volume), observed by MRI, from 1 to 4 months, on lambs from ewes with metabolic status contrasted and exposed to BPS during pregnancy

From 1 to 4 months, the lambs were monitored by pelvic MRI, to determine the size and volume of the ovaries and the number of antral follicles. No effect of any experimental group was observed on the volume nor the size of lamb ovaries (Fig. 3A and B). However, there was an effect of the age on the lamb ovary volume (*p* = 0.002), especially between lambs at 1 month (M1) and 2 months (M2) (*p* = 0.02) and M2 and 4 months (M4) (*p* = 0.002) (Fig. 3A and B).

Although no effect of the metabolic status and the BPS exposure was observed in 4-month-old lambs for the total follicle volume, exposure of mothers to BPS tend to decreased the total number of follicles per ovary per lamb (BPS effect, *p* = 0.09). The mean total follicle number per ovary per lamb were 12.7 ± 0.22 for LM0, 10.2 ± 0.09 for FM0, 7.3 ± 0.06 for LM50 and 6.0 ± 0.06 for FM50 (Table 5). Regarding the volume of follicles, 4096 were analysed in the 10 *in utero* BPS exposed lambs and 3584 follicles were analysed in the 10 control lambs. The follicles were categorised according to their volume, < 2 mm^3^, 2 mm^3^ ≤ follicle volume < 10 mm^3^ or ≥ 10 mm^3^, and to their size, 1 mm^2^ ≤ follicle size < 2 mm^2^ or ≥ 2 mm^2^, corresponding to the beginning of terminal folliculogenesis [36]. When considering the volumes of follicles, in the category of volume between 2 and 10 mm^3^, the volume tended to be reduced (*p* = 0.10) in lambs exposed *in utero* to BPS (2.69 ± 0.03 follicles) compared to control lambs (6.50 ± 0.08 follicles) (Table 5). In the category of size < 2 mm^2^, the number of follicles was significantly lower (*p* = 0.011) in lambs exposed *in utero* to BPS (2.50 ± 0.04 follicles) compared to control lambs (6.07 ± 0.06 follicles). In contrast, no significant difference was reported in follicles ≥ 2 mm^2^. The volume of the ovarian stroma and the ratio between stromal volume and ovarian volume, a predictor of ovarian androgenic dysfunction, did not significantly vary between the experimental groups, but an effect due to ovary side was observed on the ratio of stromal volume to ovarian volume (*p* < 0.0001) (Table 5).

In lamb experiment, adult ewes naturally had a contrasting metabolic status, ewes with a weight < 72 kg were classified as lean (L), and ewes with a weight > 72 kg were classified as fat (FM). The ewes received BPS in their diet of 0 g / kg / d or 50 µg / kg / d from 1 to 4 months gestation. Thus, four experimental groups were constituted (LM0, LM50, FM0 and FM50). At 1 month (M1), 2 months (M2), 3 months (M3) and 4 months (M4), the lambs (5 to 6 lambs/experimental group) were monitored for the development of their ovaries by pelvic magnetic resonance imaging (MRI), the size (mm; A.) and the volume (mm^3^; B.) of the ovaries of each lamb were determined. Results are presented as mean + SEM. Statistical analysis was performed between the experimental groups of a same age (LM0, LM50, FM0 and FM50) (A. and B.), and between age of the same experimental group (M1, M2, M3, M4). Bars with different letters are significantly different (*p* < 0.05).

**Table 5:** Population of antral follicles, observed by MRI, at 4 months, on lambs from ewes with contrasted metabolic status and exposed to BPS during pregnancy

Lamb ovarian parameters	Mean ± SEM				*p*-value		
	LM0	LM50	FM0	FM50	BPS effect	Metab effect	BPS x Metab effect
*MRI analysis*	*n* = 6 lamb	*n* = 5 lamb	*n* = 6 lamb	*n* = 6 lamb			
*Number of follicles*	12.7 ± 0.22	7.3 ± 0.06	10.2 ± 0.09	6 ± 0.06	*0.089*	0.806	0.984
*Follicle volume (mm* ^*3*^ *)*	935 ± 18.6	1109 ± 10.4	816 ± 3.57	1095 ± 25.1	0.365	0.953	0.840
*Number of follicles by volume (mm* ^*3*^ *)*							
< 2	2.33 ± 0.03	1.3 ± 0.03	2.38 ± 0.06	1.33 ± 0.03	0.125	0.935	0.993
2 ≤ Follicle < 10	7.67 ± 0.18	2.8 ± 0.05	5.62 ± 0.06	2.5 ± 0.03	*0.097*	0.954	0.974
≥ 10	2.67 ± 0.05	3.2 ± 0.04	2.25 ± 0.02	3.2 ± 0.04	0.626	0.361	0.862
*Number of follicles by size (mm* ^*2*^ *)*							
1 ≤ Follicle < 2	6.5 ± 0.08	2.4 ± 0.05	5.75 ± 0.09	2.67 ± 0.07	**0.011**	0.839	0.825
≥ 2	6.17 ± 0.15	4.9 ± 0.06	4.5 ± 0.03	3.33 ± 0.04	0.590	0.630	0.858
* Stromal volume (mm* ^*3*^ *)*	704 ± 16.1	995 ± 10.3	626 ± 4.74	966 ± 24.4	0.232	0.965	0.899
* Ratio stromal/ovary volume*	0.709 ± 0.01	0.879 ± 0.002	0.757 ± 0.004	0.734 ± 0.01	0.264	0.615	0.172

*Metab* Metabolic status, *LM0* Lean mother unexposed, *LM50* Lean mother with BPS 50 µg / kg / d, *FM0* Fat mother unexposed, *FM50* Fat mother with BPS 50 µg / kg / d

Bold text indicates significant differences (*p* < 0.05), and italicised text indicates tendencies (0.05 < *p* < 0.1)

### Plasma Anti-Mullerian hormone (AMH) in fetuses and lambs *in utero* exposed to BPS

**Fig. 4:**
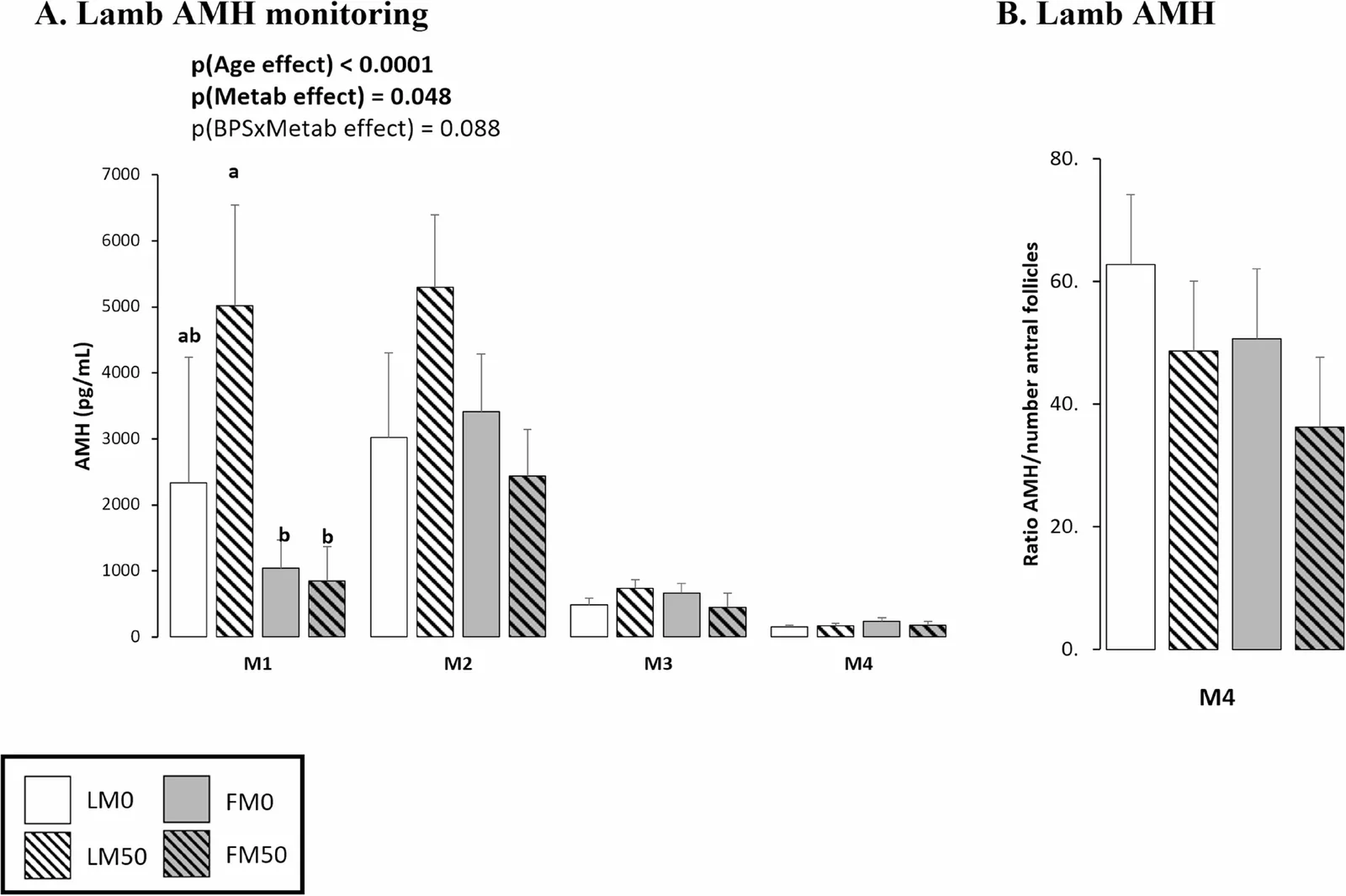
Plasma concentrations of AMH of lambs from ewes with metabolic status contrasted and exposed to BPS during pregnancy during postpartum period (A) or at sacrifice (B)

AMH was not detected in fetus plasma. Regarding the monitoring of lamb plasma, AMH level was significantly affected by the age (*p* < 0.0001) and the metabolic status (*p* = 0.048) and tended to be affected by the interaction between the metabolic status and the BPS exposure (*p* = 0.09). Indeed, the plasma AMH concentration decreased in lambs with age from M2 onwards, (M1: 2715.2 ± 743.0 pg/mL, M2: 3817.0 ± 548.4 pg/mL, M3: 614.3 ± 74.2 pg/mL and M4: 184.7 ± 22.7 pg/mL). Moreover, AMH levels were higher in LM lambs compared to FM lambs at both M1 (LM: 4072.0 ± 1199.2 pg/mL vs. FM: 940.9 ± 329.5 pg/mL) and M2 (LM: 4494.2 ± 860.9 pg/mL vs. 2994.6 ± 578.0 pg/mL), but then became similar at M3 (LM: 650.5 ± 93.9 pg/mL vs. FM: 566.8 ± 122.6 pg/mL) and M4 (LM: 163.4 ± 28.0 pg/mL vs. FM: 213.0 ± 37.3 pg/mL). Significant differences between experimental groups were only observed for M1 lambs. AMH levels were significantly lower for lambs from FM0 and FM50 compared to LM50 (*p* = 0.014, 852.8 ± 520.3 pg/mL and *p* = 0.005, 1043.8 ± 426.2 pg/mL vs. 5018.8 ± 1521.1 pg/mL, respectively) (Fig. 4A).

The ratio between plasma AMH and the total number of antral follicles is a marker of the production of AMH by follicle. No difference between groups was evidenced (Fig. 4B).

In the lamb experiment, adult ewes had a contrasting metabolic status, ewes with a weight < 72 kg were classified as lean (LM), and ewes with a weight > 72 kg were classified as fat (FM). The ewes received BPS in their diet of 0 µg / kg / d or 50 µg / kg / d from 1 to 4 months gestation. Thus, four experimental groups were constituted (LM0 *n* = 7 lambs, LM50 *n* = 11 lambs, FM0 *n* = 9 lambs, and FM50 *n* = 9 lambs). At 1 month (M1), 2 months (M2), 3 months (M3) and 4 months (M4), anti-müllerian hormone (AMH) concentration was determined in the plasma of lambs. Statistical analysis was performed between the experimental groups of the same age (M1 or M2 or M3 or M4) (A.). The plasma AMH concentration at M4 was reported to the number of antral follicles detected on MRI at 4 months (M4) (B). Results are presented as mean ± SEM. Bars with different letters are significantly different (*p* < 0.05). Metab: metabolic status.

### Effects of *in utero* BPS exposure on early folliculogenesis in fetuses and lambs

To decipher the effects of *in utero* BPS exposure on folliculogenesis, histological analyses were performed on ovary sections of fetuses (D-20) and lambs (M4), to count and categorise the follicles from the primordial to antral stage (Supplementary Fig. 2A.). The total number of follicles was not significantly influenced by the litter size neither in fetus (*p* = 0.12) nor in lambs (*p* = 0.54).

**Fig. 5:**
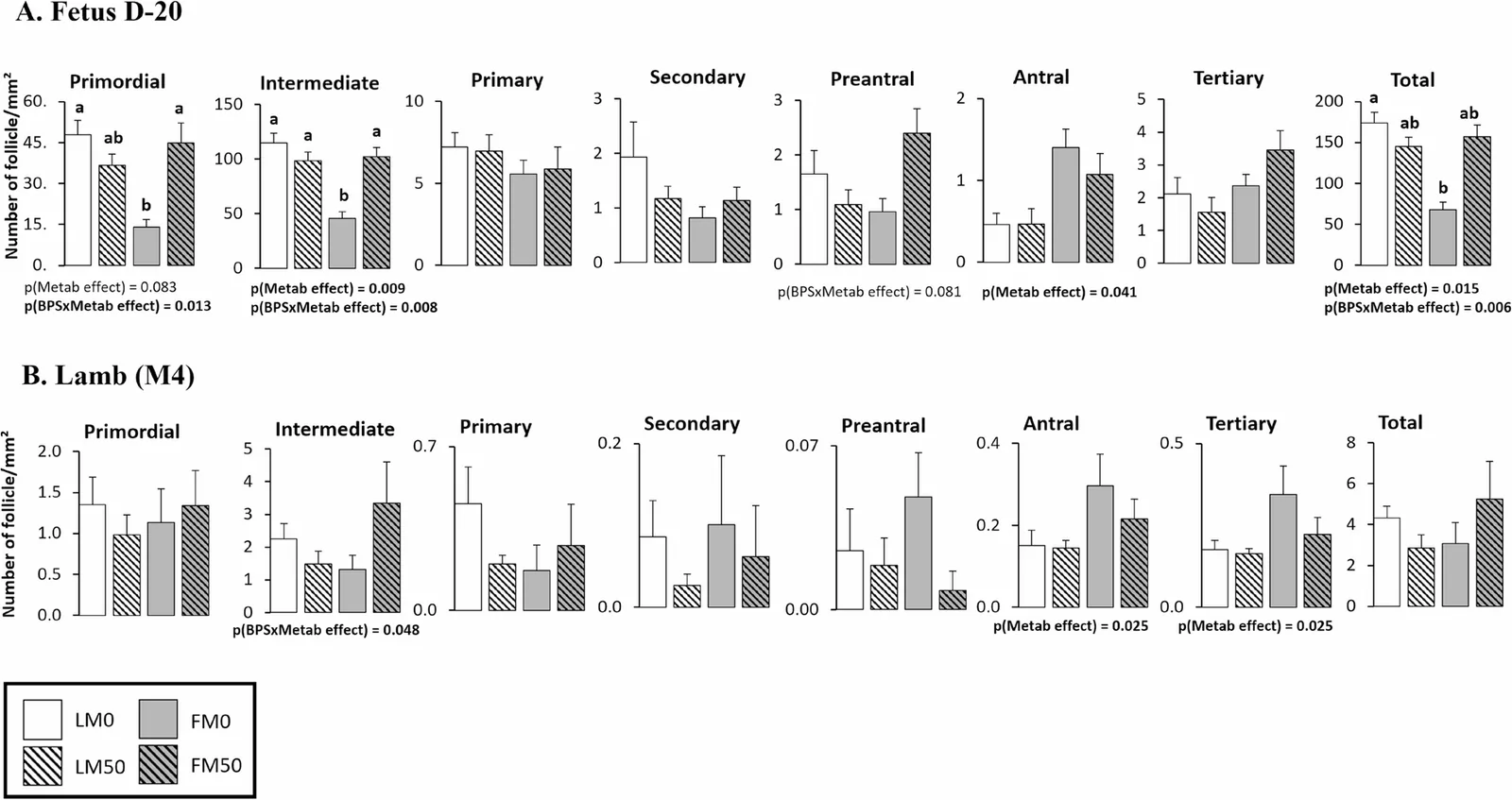
Follicular development of fetuses (A) and lambs (B) from ewes with metabolic status contrasted and exposed to BPS during pregnancy

In fetuses, the numbers of antral follicles (Metab effect, *p* = 0.041) were significantly increased in fetuses from fat mothers compared to lean ones. A significant interaction between BPS exposure and maternal metabolic status was reported for the number of primordial (*p* = 0.013), intermediate (*p* = 0.008) and the total number of follicles (*p* = 0.006), a similar trend being observed with pre-antral follicles (*p* = 0.081). Post hoc analyses revealed that the number of primordial (*p* = 0.012), intermediate (*p* < 0.001) and the total number of follicles (*p* = 0.002) significantly increased in the LM0 group compared to the FM0 group. In contrast, no significant differences related to maternal metabolic status were observed within BPS-exposed groups, suggesting that BPS exposure modulates the effect of maternal metabolic status on follicle numbers (Fig. 5A.). No difference in the atretic follicle percentage was observed between groups (Supplementary Table 1).

In lambs, the number of antral (Metab effect, *p* = 0.025) and tertiary follicles (Metab effect, *p* = 0.025) was higher in FM lambs (0.26 ± 0.05 antral follicle/mm^3^ and 0.29 ± 0.05 tertiary follicles/mm^3^) compared to LM lambs (0.15 ± 0.02 antral follicle/mm^3^ and 0.17 ± 0.01 tertiary follicles/mm^3^) (Fig. 5B.). No effect of *in utero* BPS exposure was reported. Nevertheless, a significant interaction between BPS exposure and maternal metabolic status was observed on the number of intermediate follicles per mm^2^ (*p* = 0.048), indicating that the effect of maternal metabolic status on intermediate follicle density differs according to BPS exposure (Fig. 5B).

In fetus experiment, adult ewes were fed a diet to obtain lean (LM) or fat (FM) ewes and received BPS by daily subcutaneous injection of 0 µg / kg / d or 50 µg / kg / d from 1 to 4 months gestation. Thus, four experimental groups were constituted, and among the 34 fetuses, 31 were analyzed (LM0, *n* = 7 fetuses; LM50, *n* = 8 fetuses; FM0, *n* = 9 fetuses; FM50, *n* = 7 fetuses). In lamb experiment, adult ewes had a contrasting metabolic status, ewes with a weight < 72 kg were classified as lean (LM), and ewes with a weight > 72 kg were classified as fat (FM). The ewes received BPS in their diet of 0 µg / kg / d or 50 µg / kg / d from 1 to 4 months gestation. Thus, four experimental groups were constituted and among the 36 lambs, 29 were analyzed (LM0, *n* = 6 lambs; LM50, *n* = 10 lambs; FM0, *n* = 7 lambs; FM50, *n* = 6 lambs). At 130 days of gestation (D-20) for fetuses and 4 months after birth (M4) for lambs, an ovary was fixed and embedded in paraffin for histologic analyses. The follicles from the primordial to antral stage were counted for fetuses (A.) and lambs (B.). Results are expressed as the number of follicles of each class reported on the surface of the section (mm^2^), and are presented as the mean ± SEM. Statistical analysis was performed between the experimental groups within the same follicular stage (primordial or intermediate or primary or secondary or preantral or antral or total). Bars with different letters indicate significant differences (*p* < 0.05).

## Discussion

To our knowledge, this work is the first to assess the effects of an *in utero* exposure to an endocrine disruptor associated with maternal metabolic status on ovine fetal development and until 4-month-old, using the MRI technology. In the present study, the ewe model showed that fetal weight was affected by maternal metabolic status. Maternal metabolic status and BPS exposure also disrupted plasma AMH concentration in lambs. Maternal metabolic status significantly influences fetal ovarian folliculogenesis. But BPS exposure modulates the interaction between maternal metabolic status and fetal ovarian development, consistent with an early endocrine-disrupting effect on the establishment of the ovarian follicle pool.

### AMH level and ovary weight

In ewes, the number of antral follicles remains high from birth to 2 months, then declines and stabilizes at puberty [42]. These data are consistent with plasma AMH concentrations measured in the present study in lambs. AMH is indeed secreted by the granulosa cells of the preantral and antral follicles [35]. At 2-month-old, AMH reached its maximum concentration in all the experimental groups. At 4-month-old, while none of the ovaries exhibited ≥ 25 antral follicles, AMH concentration reached its lowest level.

The AMH concentrations were higher in 1- and 2-month-old lambs born from restricted ewes, which could suggest that a lower number of follicles in lambs born from ewes with high metabolic status, but this difference seemed to regress before puberty. MRI analysis of ovarian size and volume is a good predictor of ovarian development. In the present study, no effect of the metabolic status was observed on the ovarian size and volume in lambs. Similarly, it was previously shown that the weight of the ovaries of 2-month-old lambs was not affected by a restricted diet of the ewes during the last 50 days of gestation [21]. Nevertheless, glucose regulates insulin growth factor 1 (IGF1), which has a key role in the development of reproductive organs [21, 55]. Thus, it would be interesting to determine the metabolic status of the lamb by evaluating its insulin response via plasma glucose measurements.

The observation that AMH concentrations were highest in lambs from restricted ewes exposed to BPS suggests that the BPS exposure may have a different effect depending on maternal metabolic status. It would be interesting to determine the exact number of follicles using pelvic MRI at 1- and 2-month-old lambs. In case a significant increase in the number of antral follicles is reported in the LM lambs and particularly the LM50 lambs, it could mean that the ovarian reserve would be depleted earlier compared to other experimental groups. However, no effect of metabolic status was observed on the number of follicles in 4-month-old lambs, nor on the AMH/number of antral follicles ratio detected by MRI at 4 months of age.

No effect of BPS exposure was reported on the weight of ovaries in the present study. This is in agreement with a previous study in rats in which the weight of the reproductive organs (ovary + uterus) in the offspring was unaffected by in utero BPS exposure [24].

### Follicular population

Regarding the histological data, an effect of the metabolic status of the mother was evidenced. Indeed, the offspring of lean ewes showed a lower number of antral follicles than the offspring of fat ewes. This was also observed for the number of tertiary follicles in lambs. These results are in agreement with previous experiments where undernutrition during gestation led to decreased ovulation rate of the offspring in ovine [12] and decreased antral follicle count in bovine [14]. Comparatively in mice, offspring from pregnant mother undergoing a high-fat diet also had a decrease in the number of primordial follicles at 15 weeks of life [7]. Conversely, the number of intermediate and total number of follicles decreased in fetuses from fat mothers in this experiment, while a study in rat where mothers underwent high-fat diet during pregnancy and breastfeeding shows an increase in the number of intermediate follicles in the offspring [52]. A decrease in the diameter of secondary and tertiary follicles was previously reported in ovaries of lambs of undernourished ewes during the last 50 days of gestation [21]. Thus, it would be of interest to determine whether the diameter of the same category of follicles also reduced in fetuses and lambs in the present study.

In the present study, the number of primordial follicles in fetuses and lambs was not affected by *in utero* BPS exposure. Discrepancies regarding the effect of BPS occurred in the literature. Indeed, BPS exposure was reported to lead to either an absence of effect during gestation [28], or to a decrease [47], or even to an increase in the number of primordial follicles in mice [58]. However, the number of small antral follicles (2 ≤ F < 10 mm^3^) detected on MRI tended to be lower and the number of antral follicles with a size between 1 ≤ Follicle < 2 mm^2^ was lower in 4-month-old lambs exposed to BPS compared to control lambs. This result is in agreement with studies in mice that showed a decrease in the number of antral follicles after BPS in utero or postnatal exposure [28, 58]. Such effect was not observed in rats [24], but in women, a higher urinary BPA concentration is associated with a decrease in the number of antral follicles [49]. Because the size of the ovarian reserve is reflected by the number of antral follicles [14], such decrease in antral follicles observed in lambs *in utero* exposed to BPS in the present study could suggest a premature depletion of the ovarian reserve. A low number of antral follicles leading to low progesterone and oestradiol concentrations in cattle [14, 39], it would thus be interesting to determine these hormone levels in our experiment. In lambs, at birth, the number of antral follicles is high but variable [42, 60]. Therefore, significant effects of BPS exposure and / or maternal metabolic status could be masked by this inter-individual variability. In order to confirm our results, a greater number of animals should be examined. Lastly, in 4-month-old lambs, the ratio of ovarian stromal and total ovarian volumes was not modified between the experimental groups. This ratio is a predictor of androgenic dysfunction [60]. Thus, maternal metabolic status and / or *in utero* BPS exposure should not interfere with ovarian androgen secretion. Despite an absence of significant difference regarding the atretic follicle percentage, a tendency of a group effect was observed on primordial follicle atretic percentage. A future study could focus on this question to decipher whether BPS have a potential effect on follicle atresia.

The present study reported that the effects of BPS exposure on the follicle population depends on the body condition of the animal. The modulation of metabolic status–dependent effects by maternal exposure to BPS has previously been reported in the ovarian function of adult ewes. Indeed, a significant interaction between BPS exposure and the metabolic status of the ewes occurred on the number of cleaved oocytes and blastocysts that developed under in vitro fertilization (IVF) [10], on the follicular fluid steroidome [51] and on the granulosa cell proteome [27].

A significant interaction between BPS exposure and maternal metabolic status was reported on the number of primordial, intermediate and total follicles in fetuses and on the number of intermediate follicles in lambs. In a similar study where lambs were exposed to 50 µg/kg/day BPA during early postpartum by subcutaneous injections, an increase in the number of intermediate follicles was also reported [44]. Further studies are needed to determine whether the decrease in the number of primordial follicles observed in the FM0 group is a consequence of a lower proliferation and / or of a higher depletion of follicles.

Interestingly, two lambs that had been exposed to BPS *in utero*, one LM50 lamb and one FM50 lamb, exhibited corpora luteum on their ovaries meaning they already ovulated. Although it is possible to observe antral follicle early in life, puberty in ewes with the first ovulation does not appear until 7–8 months [42]. Future studies could investigate the effect of *in utero* BPS exposure on the precocity or the onset of puberty.

### Offspring weight and development

The fetuses of the lean ewes exhibited a lower weight (-10%) compared to the fetuses of the fat ewes. Similar results were reported with a -17% reduction in weight at 130 days of gestation in fetuses of young under-fed ewes [29]. Such difference in weight due to maternal metabolic status was not observed in lambs, in the present study. A restricted maternal diet leads to a restriction of fetal growth associated with alterations in maternal placental vascular development [30], as well as reduced maternal and fetal plasma glucose (-16% and − 45% respectively) [29]. Thus, it would be interesting to assess whether lambs develop insulin resistance by evaluating their glycemic response to insulin in fetuses from either lean or fat ewes.

BPS *in utero* exposure has no effect on the body weight of lambs in the present study. However, it was previously reported that BPS induced lipid accumulation, differentiation of primary human pre-adipocytes [2] exacerbated adipogenesis in mice *in utero* exposed [1] and is correlated with body mass index (BMI) in human [23]. Differences in fetal growth can have long-term developmental and metabolic consequences [29]. It is thus possible that the obesogenic effects of bisphenols do not appear until puberty [1, 20, 41].

No effect of BPS exposure or maternal metabolic status was observed on the normalised organ weight in female lambs and on fetus and lamb anogenital distance during this experiment. Nevertheless, a birth cohort study highlighted that the effect of bisphenol on anogenital distance was not consistent. Indeed, at 6 months, boys whose mothers had been detected with BPA and its analogues BPF, BPS, and BPAF had a longer AGD, while at 12 months, an inverse association was observed for BPA, BPS, and BPAF [6]. Other studies have shown that BPA *in utero* exposure of male fetuses can reduce this anogenital distance in human and mice [22, 45]. Conversely, anogenital distance in female mice was longer at 22 days, but with no difference at 60 days compared to controls [22]. Anogenital distance in girls in utero exposed to bisphenols were mainly observed at 48 months [6]. It might therefore be interesting to conduct a longitudinal analysis over several months for this parameter, since it appears that this distance is not constant over time. Furthermore, measuring anogenital distance in females is more difficult because this distance is shorter, consequently small measurement errors can therefore have a proportionally greater impact. In addition, anogenital distance in females shows minimal variation over time, which limits the sensitivity for detecting early effects [16].

### Gestation, BPS exposure and metabolic status

The exposure to BPS from 1 to 4 months gestation in lean or fat pregnant ewes did not alter the gestation duration and the number of fetuses per litter. Few studies showed discrepancies regarding the relationship between exposure to bisphenols and gestation duration. In the present study, no risk of preterm birth was reported following bisphenol exposure, which is relevant with a recent human study [48]. It was suggested that women BPS exposure may extend pregnancy duration for girls only [53]. However, a modelisation study suggested that women BPS exposure could lead to an increased risk of preterm delivery [59]. Further studies are needed in order to accurately determine the effects of BPS on the gestation length. Nutritional limitation of young ewes during gestation was not associated with premature birth [29], which is also the case in the present study. Although the number of male lambs per litter tends to decrease in ewes exposed to BPS, there is no significant difference in the sex ratio, which is in agreement with studies on rats exposed to BPS at 5 µg/kg/day or on mice with high-fat diet during gestation [7, 24].

### Advantages and limits of the study

This study combined two separate and complementary experiments on fetuses and new-born lambs, sharing a common goal. In both experiments, the same breed of sheep, with ewes originated from the same herd (INRAE Experimental Unit PAO, Nouzilly, France), therefore with a same genetic background, was selected as a relevant toxicology model for human reproduction. In fact, some reproductive characteristics of ewes of this breed (Ile-de-France) are similar to human ones with generally 1 to 2 offspring per gestation, and in that the ovarian development takes place during fetal life (unlike in rodents) [5, 41]. In addition, the sheep model was used to predict human fetal exposure to BPA [8] and BPS [18]. The sheep therefore is an excellent model to investigate the relationship between maternal metabolic status [5, 12, 41] and exposure to bisphenols [15, 19, 44, 46] on the reproductive function of offspring.

The diet designed for the ewes reached its intended purpose. Indeed, ewes that were fat at the beginning of the experiment maintained higher weights than lean ewes throughout the whole experimental period. This had already been observed in our previous studies in which ewes were fed a similar diet [10, 27, 51]. Furthermore, BPS-g was only observed in exposed ewes, compared with unexposed ewes. As expected, fetal weight was influenced by the metabolic status of the mother as well as by the number of fetuses per litter. In addition, lamb growth, especially the weight and volume of organs such as the ovaries, is positively correlated with the age of the individuals, and also with litter size but no effect of the mother metabolic status is observed. The experimental design included artificial rearing because the objective was to affect only the gestational period in terms of both metabolic environment and BPS exposure. Therefore, after lambing, all lambs were conducting on artificial rearing, to the exceptions of the first meals to ensure lamb viability. Nervertheless, it is true that natural maternal feeding is an important regulator of neonatal developmental programming and artificial milk feeding might influence the ovarian follicle development.

In the fetal experiments, the pregnant ewes received BPS 50 µg / kg / day in daily subcutaneous injections adjusted to their weight, allowing an evaluation of BPS effects on fetal development at the regulatory dose set for BPA in 2015 [13]. Because food is the main source of human exposure to BPS [54], in the lamb experiment, the pregnant ewes were exposed to BPS at a dose of 50 µg / kg / day via the diet. In addition, this exposure to BPS lasted from 1 month to 4 months of gestation, therefore covering the critical window of organogenesis. Moreover, the characterization of BPS effects started in 1-month-old lambs, therefore 2 months after the end of BPS exposure. This experimental design therefore highlighted the persistent effects of BPS on the lamb after birth. It was not done in the present study, but it would be really interesting to investigate the maternal endocrine milieu to assess whether it contributed to the observed alterations in ovarian development during the prenatal period.

Because BPS effects or significant interaction between BPS exposure and maternal metabolic status were observed up to 5 months after the end of the exposure, future studies should focus on epigenetic investigation of follicular cell methylome of several categories of follicles (i.e. granulosa cells). Epigenetic effect of BPS was previously reported in mice liver [4]. However, during fertilization, the embryonic genome undergoes demethylation, followed by *de novo* methylation at the blastocyst stage. At this stage, the maternal environment could thus modify the expression of fetal genes, thereby affecting offspring health, but also of subsequent generations.

One limitation of the lamb experiment is that the pregnant ewes were housed and fed in experimental groups and not individually. Nevertheless, BPS-g levels showed that all ewes were similarly exposed to BPS through the diet. In addition, plasma BPS-g of the ewes of the two experiments were carried out at different times after BPS oral/subcutaneous administration (1 h vs. 24 h), making direct comparisons of exposure levels difficult. However, considering differences in bioavailability, it is highly likely that ewes from the fetus experiment, exposed by subcutaneous injections, have higher exposure levels than ewes from lamb experiment exposed through diet [17]. Nevertheless, similar profile of ovarian alterations was observed in both experiments. Despite a substantial number of animals included in these experiments, the results should be confirmed with larger groups, because of several variation factors (litter size, sex ratio). Only a single dose of BPS has been tested (the tolerable daily intake set for BPA), even though this endocrine disruptor could exhibit non-linear dose-response effects. It would be interesting to evaluate the alterations caused by lower doses. Furthermore, MRI analysis of the follicles was only performed at 4 months of age, since this assessment is very time-consuming.

This study aimed at investigating the effects of *in utero* BPS exposure and metabolic status on fetal and early postnatal development. Unfortunately, this experimental design did not allow follow up until puberty or adulthood, which could have been interesting. Indeed, previous work has shown that fetal exposure to BPS (50 µg/kg/d), from gestation day 9 to birth in mice, altered reproduction later in life, such as difficulties in maintaining a pregnancy [47]. Thus, further studies will be needed to determine whether these alterations in folliculogenesis observed before puberty will also affect the reproductive performance of adult animals.

## Conclusion

The maternal environment, combining BPS exposure and maternal contrasted metabolic status, affected the establishment or depletion of ovarian reserve, which could have long-term consequences on folliculogenesis in pre-pubertal and adult animals. Importantly, the effect of BPS differed according to the maternal metabolic status. Thus, the conclusions on the BPS effects on ovarian and follicular development should be interpreted in function of the maternal metabolic status. This status should indeed be considered for future research on the effects of bisphenols. Nevertheless, the mechanisms underlying the interaction between BPS exposure and maternal metabolic status on in utero ovarian development of young female lambs remain to be explored.

## Supplementary Information

Supplementary Material 1.

Supplementary Material 2.

## Data Availability

Data available on request from the authors.
