# Supplementary material for: Bisphenol S fetal exposure in ewes exhibiting contrasted metabolic status impaired the ovarian follicular development of fetuses and lambs
Source: J Ovarian Res. 2026 Jun 30;19:290. doi: 10.1186/s13048-026-02182-y (PMC13587282; doi:10.1186/s13048-026-02182-y)
Supplement: Supplementary file 1 [file 13048_2026_2182_MOESM1_ESM.pdf]

Supplementary Figures :

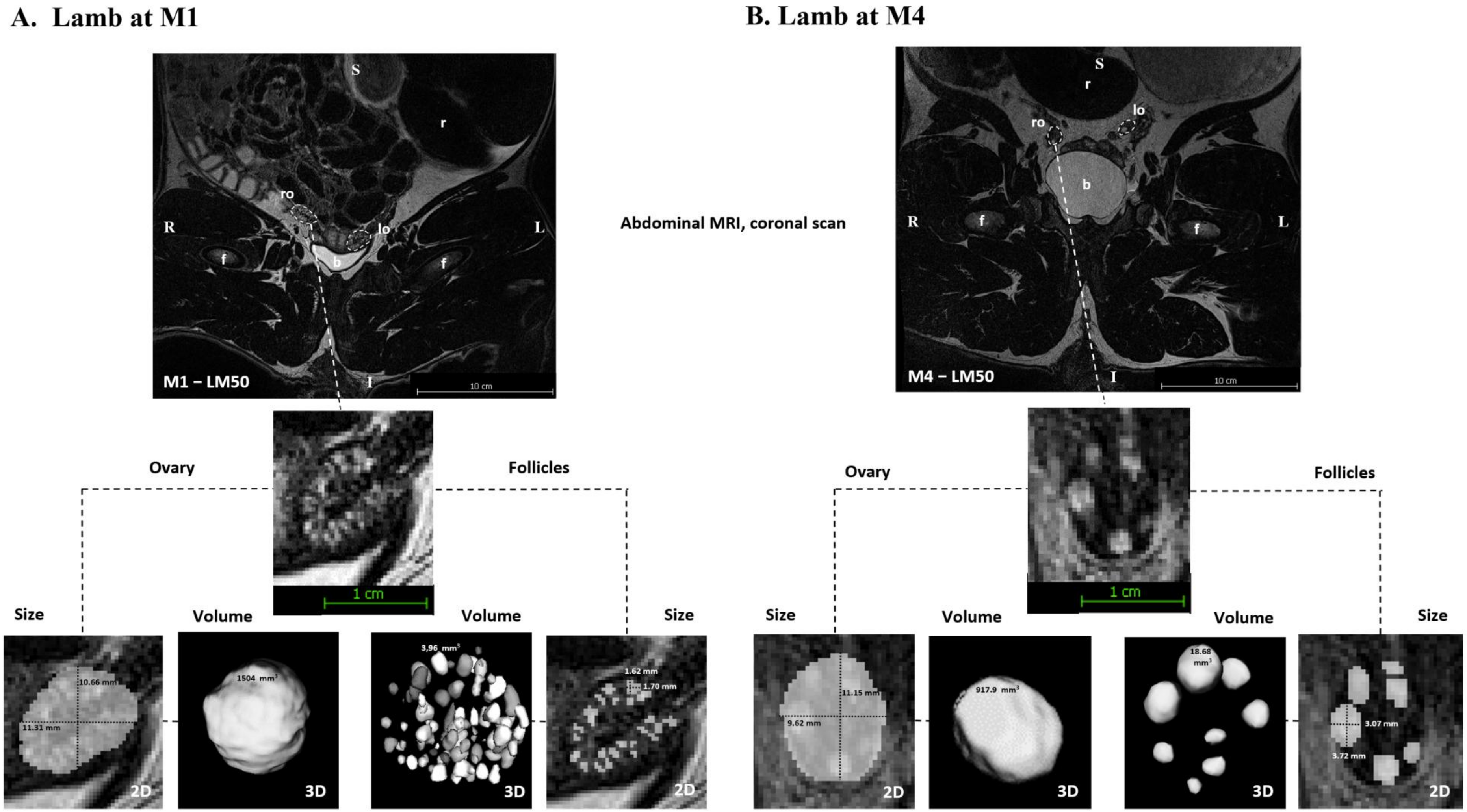

Supplementary Figure 1: Analyses of ovarian development and antral follicle population of lambs from MRI acquisition

After birth, the lambs underwent a pelvic MRI scan. On coronal axis, we distinguish the ovaries circled in white dotted lines (right ovary (ro) and left ovary (lo); femur (f), rumen (r), bladder (b)). With the ITK-SNAP software (www.itksnap.org), we obtained 2D and 3D images. Thus, we were able to follow ovarian development (size and volume) and the population of antral follicles ≥ 1mm (number, size and volume), which correspond to white ovoid structures. This analysis was carried out at 1 month (A.), 2 months, 3 months and 4 months (B.) on 5 lambs per experimental group. L: left, R: right, S: superior, I: inferior.

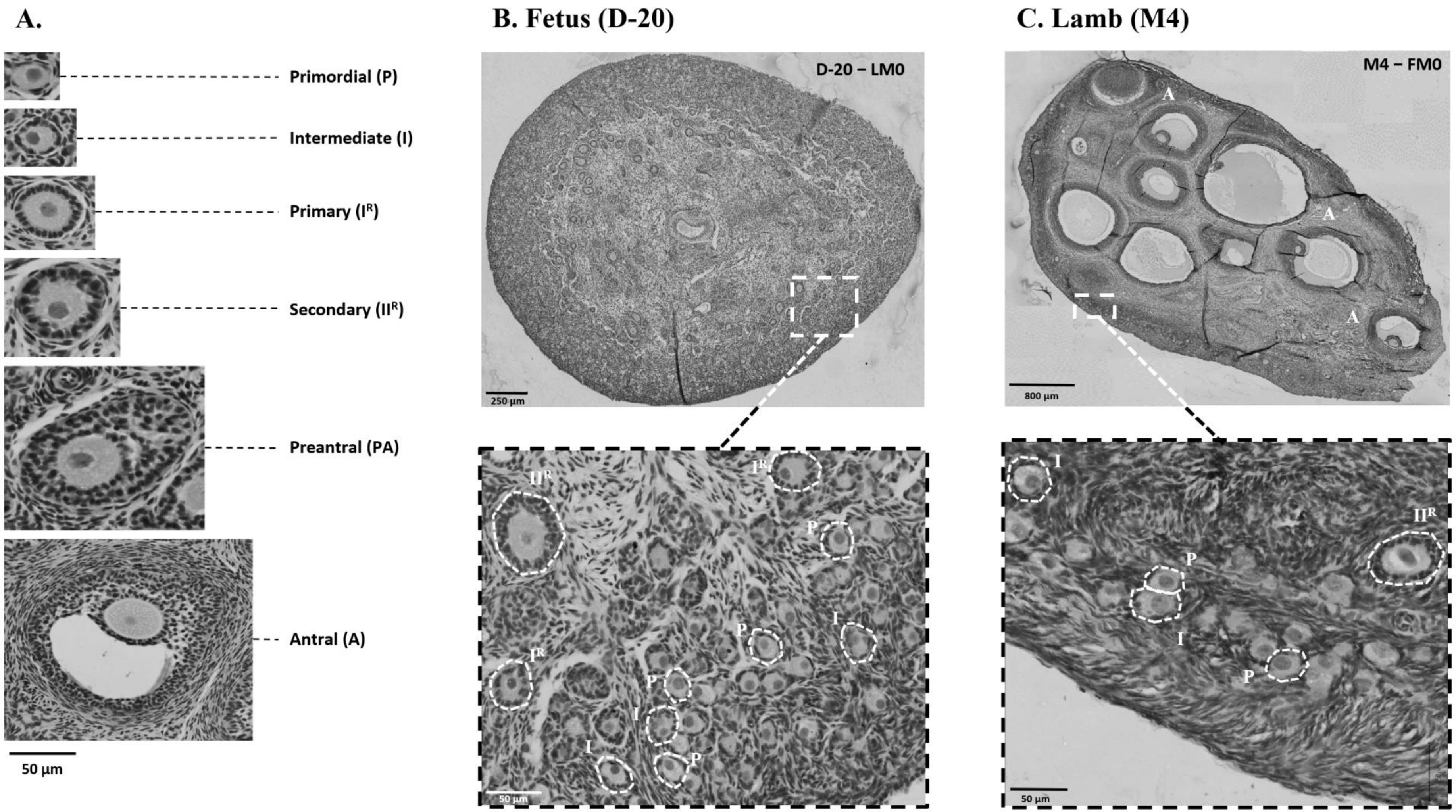
A.
Primordial (P)
Intermediate (I)
Primary (IR)
Secondary (IIR)
Preantral (PA)
Antral (A)
50 µm
B. Fetus (D-20)
D-20 – LM0
250 µm
50 µm
C. Lamb (M4)
M4 – FM0
800 µm
50 µm

Supplementary Figure 2: Follicular population of fetuses and lambs in ovarian histology

One ovary from each fetus (D-20) and lamb (M4) was fixed with 4% paraformaldehyde, embedded in paraffin, serially sectioned to a thickness of 7 µm with the microtome and slides were stained with hematoxylin. Slides were scanned and with QuPath-0.2.3 software (University of Edinburgh, United Kingdom; https://qupath.github.io/), the follicles were classified based on the shape and number of somatic cell layers surrounding the oocyte, as well as the presence or absence of an antrum. Thus, the follicles were organized into six classes: primordial follicle with only granulosa cells (GC) flattened around the oocyte, intermediate follicle with at least one GC rounded around the oocyte, primary follicle with a layer of GC rounded around the oocyte, secondary follicle with two layers of GC, pre-antral follicle with more than two layers of granulosa cells, and antral follicle with the presence of an antrum (A.). 31 fetus ovaries (n = 9-11/experimental group; B.) and 35 lamb ovaries (n = 8-10/experimental group; C.) were analyzed.
