# Supplementary material for: Bisphenol S fetal exposure in ewes exhibiting contrasted metabolic status impaired the ovarian follicular development of fetuses and lambs
Source: J Ovarian Res. 2026 Jun 30;19:290. doi: 10.1186/s13048-026-02182-y (PMC13587282; doi:10.1186/s13048-026-02182-y)
Supplement: Supplementary file 2 [file 13048_2026_2182_MOESM2_ESM.pdf]

Supplementary Table 1 : Comparison of the percentage of atretic follicles in each class between groups

n fetus	7	7	7	5	ANOVA
groups	LM0	LM50	FM0	FM50	Group effect
n primordial follicle	311	453	384	440	p-value
% atretic primodial follicle	16.2 ± 2.4	8.4 ± 0.9	13.1 ± 2.5	11.9 ± 1.1	0.061
n intermediate follicle	635	1079	787	781	
% atretic intermediate follicle	23.4 ± 2.6	19.3 ± 1.5	23.6 ± 2.4	17.1 ± 1.5	0.13
n primary follicle	42	72	57	62	
% atretic primary follicle	6.8 ± 5.3	2.5 ± 1.6	9.0 ± 4.1	9.7 ± 3.6	0.54
n secondary follicle	7	13	15	9	
% atretic secondary follicle	6.2 ± 6.2	7.1 ± 7.1	22.2 ± 11.1	9.7 ± 6.2	ND
n preantral follicle	13	11	20	14	
% atretic preantral follicle	21.5 ± 10.8	0.0 ± 0.0	2.8 ± 2.8	9.0 ± 5.6	ND
n antral follicle	2	4	7	12	
% atretic antral follicle	0.0 ± 0.0	0.0 ± 0.0	33.3 ± 33.3	4.8 ± 4.8	ND
n total follicle	1010	1632	1270	1318	
% atretic total follicle	20.4 ± 2.2	15.8 ± 1.1	19.5 ± 2.1	14.7 ± 1.2	0.096

ND: not determined because the number of atretic follicle was too low (<5) in some conditions
